# Advancing the study of solving linear equations with negative pronumerals: A smarter way from a cognitive load perspective

**DOI:** 10.1371/journal.pone.0265547

**Published:** 2022-03-18

**Authors:** Bing H. Ngu, Huy P. Phan

**Affiliations:** School of Education, University of New England, Armidale, Australia; French National Center for Scientific Research (CNRS) & University of Lyon, FRANCE

## Abstract

Central to *cognitive load theory* is the concept of *element interactivity*, which reflects the complexity of material. The complexity of linear equations depends on the number of operational and relational lines and the nature of the operation (balance *versus* inverse) in the solution procedure. A relational line refers to the quantitative relation whereby the right-hand side of the equation equals to its left-hand side. An operational line refers to the application of an operation and such a procedural step preserves the equality of the linear equation. The balance method and inverse method differ in the operational line (e.g., + 3 on both sides vs.– 3 becomes + 3) where the inverse operation imposes half the level of element interactivity as the balance method. Seventy-five students randomly assigned to either the balance group or inverse group to complete (i) one-step equations (Experiment 1), (ii) two-step equations (Experiment 2), and (iii) one-step and two-step equations with a focus on equations with negative pronumerals (Experiment 3). Performance favoured the inverse group when the gap between the low and high element interactivity equations was substantial enough. Both groups performed better and invested lower mental effort on the inverse operation than the balance operation.

## Introduction

Researchers have identified the challenge associated with manipulating negative number for both adult and children [1–3]. Regarding the topic of *linear equations*, which is the focus of this study, researchers have identified a cognitive gap in understanding the difference between a negative number (e.g.,– 7) and a negative pronumeral (e.g.,– 7*x*), for example:– 7*x* = 14 [4]. Such difficulty was reflected in student learning experience of linear equations that involve both negative numbers and negative pronumerals [5–7].

**Fig 1:**
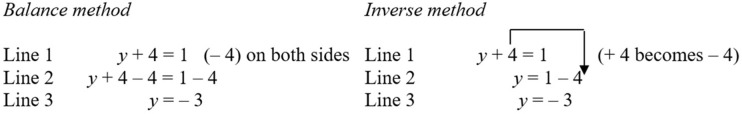
The balance method and inverse method to solve a one-step equation.

In light of student difficulty in manipulating negative numbers and negative pronumerals, how can mathematics educators enhance student learning of linear equations that involve a negative pronumeral? Many mathematics educators have advocated the use of the *balance method* (Fig 1), which is the popular method for learning to solve linear equations [8]. To the best of our knowledge, apart from our prior studies [e.g., 9], no published study has recommended alternative methods for learning to solve linear equations–for example, in our case, the *inverse method*. The present study intends to continue our research in examining the balance method and the inverse method for learning to solve linear equations with a particular focus on linear equations that involve a negative pronumeral. Our main research question then is: *can the inverse method overcome the inherit difficulty of learning to solve linear equations that involve a negative pronumeral*?

Our prior studies have compared the balance method and inverse method for learning to solve linear equations from the perspective of cognitive load [e.g., 9–11]. The main difference between the balance method and the inverse method lies in the procedural step (Fig 1, Line 2). The inverse operation (+ 4 becomes– 4), in this case, imposes lower cognitive load than the balance operation (– 4 on both sides), given that the interaction of elements occurs on one side and two sides of the equation, respectively.

In one of our studies [9], we randomly assigned middle school students to the balance method (*n* = 36) or the inverse method (*n* = 35) for learning to solve one-step and two-step linear equations. They completed a pre-test, an acquisition phase and a post-test. The inverse group outperformed the balance group for two-step equations but not one-step equations. In addition, the inverse group scored higher than the balance group for individual linear equations that had a negative pronumeral (e.g., 6 –*q* = 10) rather than a positive pronumeral (e.g., *y* + 3 = 1). In another study, using the same experimental design, the inverse group (*n* = 15) outperformed the balance group (*n* = 14) for learning to solve multi-step linear equations (e.g, 5*x* – 7 = 2*x* + 11) [10]. More recently, we examined Australian (*n* = 38) and Malaysian (*n* = 38) pre-service teachers’ ability to solve one-step, two-step and multi-step linear equations [11]. An analysis of the solution strategies revealed that the Australian and Malaysian pre-service teachers used the balance method (except one who used the inverse method) and inverse method, respectively. The Malaysian pre-service teachers outperformed Australian pre-service teachers across one-step, two-step and multi-step equations. For the concept test, both Australian and Malaysian pre-service teachers performed better for the inverse operation than the balance operation. Overall, interestingly, the findings of our prior studies were in favor of the inverse method. We accounted two possible reasons for this observation, namely: (1) differential *complexity* of linear equations, and (2) the *pedagogical method* used to solve linear equations (balance method *vs*. inverse method).

The present study, in part, expands on our previous undertakings but differs from previous studies in a number of ways: (i) performance outcomes included not only procedural knowledge but also conceptual knowledge, (ii) the use of varying solution steps in solving linear equations with a negative pronumeral (i.e., applied two inverse operations not only sequentially but also concurrently), and (iii) a measure of cognitive load in judging the balance operation and inverse operation. We will begin with the discussion of cognitive load theory that underpins the present study.

### Cognitive load theory

Cognitive load theory has provided theoretical insights to assist educators to design various instructions for effective learning [12]. Central to cognitive load theory is the human cognitive architecture that comprises a long-term memory and a working memory. The long-term memory stores domain-specific knowledge in the form of schemas [13]. The working memory is constrained in both capacity and duration. According to Miller [14], it can process about seven units of information, and Cowan [15] suggested that it can process about four units of information. Moreover, information will be readily lost from memory if it is not rehearsed [16]. However, the restriction of working memory when it deals with unfamiliar information disappears when it deals with familiar information in the form of schemas that can be retrieved from the long-term memory. The characteristic of working memory to process a schema as a single unit of element instead of multiple interactive elements reduces cognitive load. Accordingly, cognitive load researchers seek to design instructions to minimize overloading working memory in order to facilitate the acquisition of schemas, which will be transferred and stored in the long-term memory.

Recent development of cognitive load theory revisits the importance of *element interactivity*, which acts as an index to indicate the complexity of the unit material [17]. The level of element interactivity of a unit material depends on the number of elements, and the extent to which individual elements interact. Anything that requires learning constitutes an element (e.g., a symbol, a number, a concept, etc.) [18]. Sweller [17] regards element interactivity as a common thread that exists across three types of cognitive load:

- *Extraneous cognitive load* occurs as a result of suboptimal instructional design. Investing cognitive resources to process element interactivity that impedes learning constitutes extraneous cognitive load.
- *Intrinsic cognitive load* emphasizes the investment of cognitive resources to process element interactivity, which arises from the inherent complexity of the unit material. The intrinsic cognitive load depends on both the level of element interactivity of the learning material and the learner levels of expertise. There is an inverse relationship between intrinsic cognitive load and learner expertise. Once multiple interactive elements are learnt, they can be “chunked” into a schema, which reduces intrinsic cognitive load [19]. Accordingly, we can change intrinsic cognitive load by changing either the level of element interactivity of the learning material or the learner levels of expertise. In the present study, differential level of element interactivity of the learning material occurs consequently as a result of the manner in which the linear equations were presented to students (balance method *vs*. inverse method).
- *Germane cognitive load* is concerned with the use of cognitive resources to process element interactivity, which is intrinsic to the learning material. Thus, germane cognitive does not exert an independent source of cognitive load; rather, it is part of the intrinsic cognitive load. For example, requiring learners to distinguish a similar solution across variable contexts increases germane cognitive, which improves learning outcomes [20]. It should be noted that only more knowledgeable learners would benefit from high variability materials. Having discussed the three types of cognitive load in terms of element interactivity, we will discuss element interactivity and understanding in mathematics education in the next section.

### Element interactivity and understanding

In mathematics education, learning to recognize individual numbers (e.g., 6) is regarded as a low element interactivity task because a student can learn how to recognise one number independent of another number. In contrast, learning to solve a linear equation such as *x*– 7 = 10 constitutes a high element interactivity task. The learner not only needs to know individual elements (i.e., *x*,– 7, =, 10), but also the relation between them. He or she needs to know the meaning of *x*, the quantitative relation between the right side and left side of the equation, and the application of an operation such as + 7 on both sides (balance method) to solve the equation. Simultaneously coordinating the relationship between these multiple interactive elements in working memory to allow understanding to occur would impose a high cognitive load. Nevertheless, a mathematics teacher can process multiple interactive elements as a single element (a schema) with minimum cognitive load owing to his or her expertise in algebra.

In the present study, similar to our prior studies [9], we used the concept of element interactivity to explain the complexity of a linear equation (e.g., a linear equation with a negative pronumeral, 3 – 2*x* = 10), as well as the complexity of an instructional method (balance method *vs*. inverse method) that stems from different ways in presenting the solution procedure of a linear equation. An instructional method that incurs a higher level of element interactivity would be less effective owing to the imposition of higher cognitive load and vice versa. Before we detail the difference between the balance method and the inverse method to facilitate learning of linear equations from an element interactivity perspective, we will discuss prior research on the balance method and the inverse method in relation to linear equations.

### Balance method and inverse method of learning

Mathematics educators tend to use a *balance scale* to illustrate the ‘ = ‘ sign concept [6, 21], which is central to equation solving. The balance scale is effective in demonstrating the ‘take away’ of the same quantity (e.g., two marbles) from both sides in order to preserve the equality of the balance scale. Connecting concrete items (e.g., unit block) in the balance scale and symbolic equations (e.g., *x* + 7 = 12) enabled learners to develop analogical reasoning between the balance scale and the concept of equality in equations [22]. However, it is impossible to use the concept of ‘take away’ to remove a negative number or a negative pronumeral from both sides of the balance scale [6]. Moreover, Pirie and Martin [23] argued that the balance model itself creates errors when subtracting a negative number from both sides of the equation. A recent review by Otten, Van den Heuvel-Panhuizen, and Veldhuis [24] indicated that the balance model is a rather complex tool, and researchers have not identified the conditions under which it would work effectively for linear equations.

Despite the issues surrounding the use of the balance scale to scaffold negative numbers and negative pronumerals, mathematics educators have modelled the balance scale to generate the *balance method* for teaching and learning of linear equations (see Fig 1) [8]. For example, Linchevski and Herscovics [7] demonstrated the decomposition of a larger term (number, pronumeral) on one side of the equation, and then the cancellation of identical terms on both sides of the equation to maintain the equality of the equation. Interestingly, having exposed to the balance method, two top students generated a short-cut version of the balance method by using the inverse operation to solve subsequent linear equations. In their study, Andrews and Sayers [25] reported that a teacher from Finland introduced the concept of equality in the context of linear equations to grade 8 students using the balance model. Subsequently, the teacher summarized the solution procedure with an emphasis on ‘change side, change sign’, which is a short-cut version of the balance method.

Mathematics education researchers have criticized the application of the *inverse method* for solving linear equations that relies on the concept of ‘change side, change sign’ [26]. This criticism may stem from the fact that many students, in general, fail to articulate and/or understand how the inverse method actually works. For example, consider 3*x* + 5 = 17; students may mechanically pick a term (e.g., + 5) as the first step, and perform “change side, change sign” to obtain 3*x* = 17–5. They may fail to conceptualize– 5 as inverse to + 5 when applying the inverse operation to solve the linear equation. However, a review by Cai, Lew, Morris, Moyer, Ng, and Schmittau [27] indicates that South Korea, Singapore and China have introduced the *inverse operation* in elementary mathematics education. In China, for example, teachers introduce subtraction as the inverse operation of addition. To guide students to find ‘ ( )’ in 2 + ( ) = 6, the concept of subtraction is introduced: 6–2 = 4.

Mathematics education researchers have acknowledged the limitation of the balance model to scaffold the role of the ‘ = ‘ sign when dealing with linear equations that involve a negative number and a negative pronumeral [3]. Nevertheless, they used the balance method to highlight the role of the ‘ = ‘ sign when solving linear equations. On the other hand, they tended to view the inverse method as a means to perform ‘change side, change sign’, which does not adequately address the role of the ‘ = ‘ sign concept [26]. In the present study, we focused on the characteristics and comparative effectiveness of the balance method and the inverse method to facilitate learning of linear equations. More specifically, we wished to identify and validate the operational functioning of the balance method and the inverse method of learning one-step and two-step equations, which involve varying levels of complexity (e.g., a negative pronumeral). Furthermore, as we discuss, cognitive load theory [12] can facilitate understanding in regard to the effectiveness of a particular instructional approach for learning.

### Element interactivity, balance method, and inverse method

One important characteristic that differentiates the balance method and the inverse method of the procedure in equation solving is the *complexity of relational and operational lines* [9, 10, 28, 29]. A relational line describes a relation, thereby the left side of the equation equals to its right side. An operational line, in contrast, requires the use of an operation to change the state of the equation, and such procedural step preserves the equality of the equation. Fig 1 illustrates a one-step equation with a positive pronumeral. As indicated, the balance method and the inverse method share similar relational lines (Line 1 and Line 3), but they differ in the operational line (Line 2). The element interactivity associated with the balance method and the inverse method will be discussed next.

#### Balance method

Line 1 involves six elements: a pronumeral (*y*), two numbers (+ 4, 1) and three concepts. As noted earlier, a concept is considered as an element because it requires learning [18]. The three concepts are: (i) the ‘ = ‘ sign indicates a relation thereby the right of the equation equals to its left side, (ii) the number 4 is added to *y*, and (iii) to solve for *y*, the learner performs the same operation on both sides of the equation in order to maintain its equality. Line 2 involves seven elements: a pronumeral (*y*), four numbers (4,– 4, 1,– 4) and two concepts. These two concepts are: (i) cancel + 4 with– 4 on left side, and (ii) perform 1–4 on the right side in order to preserve the equality of the equation. The interaction between– 4 and the respective elements occurs on both sides of the equation. Line 3 involves three elements: a pronumeral (*y*), one number (– 3) and one concept. This concept involves: once Line 1 and Line 2 has been successfully processed, the learner would know that *y* equals to– 3 is the solution.

#### Inverse method

The balance method and the inverse method differs in the operational line (Line 2). The inverse operation conceptualizes, for example, the notion that addition is inverse to subtraction. A learner would move + 4 from the left side of Line 1 to become– 4 on right side of Line 2 in order to maintain the equality of the equation. On the right side of the equation, interaction between elements occurs thereby– 4 interacts with 1.

To enable understanding to occur, the learner needs to simultaneously coordinate multiple interactive elements within and across one operational and two relational lines for both the balance method and the inverse method. Differential level of element interactivity occurs in the operational line, in this case, favours the inverse method. Specifically, element interactivity occurs on the right side of the equation for the inverse method (i.e.,– 4 interacts with 1), but on both sides of the equation for the balance method (i.e., + 4 interacts with– 4 on left side, and 1 interacts with– 4 on the right side).

Nevertheless, the balance method was not inferior to the inverse method for one-step equations with a positive pronumeral because the total cognitive load needed to process the level of element interactivity would have been low, regardless of the balance method or the inverse method [9]. In contrast, regarding one-step and two-step equations with a negative pronumeral (Appendix A in S1 Appendix) that have higher number of operational and relational lines, we expect differential level of element interactivity would favor the inverse method.

### One-step and two-step equations with a negative pronumeral

How we apply the inverse operation influences the level of element interactivity between the balance method and the inverse method for one-step and two-step equations. Regarding one-step equations with a negative pronumeral (e.g., 8 –*a* = 13), for example, we can apply two inverse operations sequentially or concurrently (Appendix A in S1 Appendix). Here, a learner can apply two inverse operations sequentially, resulting in two operational and three relational lines. Thus, from this, any advantage of the inverse method over the balance method may disappear, given that they have similar number of operational and relational lines.

The flexibility of the inverse operation allows the use of two inverse operations concurrently instead of sequentially. For one-step equation with a negative pronumeral such as 11 –*x* = 7 (Appendix A in S1 Appendix), applying two inverse operations concurrently (–*x* becomes + *x* and + 7 becomes– 7) in a single operational line resulted in one operational and two relational lines. Consequently, differential level of element interactivity would favor the inverse method because it has one less operational line (2 *vs*. 1) and relational line (3 *vs*. 2). Moreover, applying two inverse operations concurrently also allows the learner to operate with a positive pronumeral. Therefore, the inverse method is advantageous only when we use two inverse operations concurrently.

In regard to two-step equations with a negative pronumeral such as 22 – 5*p* = 2 (Appendix A in S1 Appendix), applying two inverse operations sequentially results in the inverse method and the balance method sharing two operational and three relational lines, thus indicating no differential benefit favoring the inverse method. However, the inverse method is advantageous over the balance method because of the differential level of element interactivity that exists for each operational line (e.g.,– 22 + 22 – 5*p* = 2–22, balance operation *vs*.– 5*p* = 2–22, inverse operation). With respect to each operational line, the ratio of the number of the interactive elements between the balance operation and the inverse operation is 2:1. Nevertheless, applying two inverse operations concurrently enables learners to operate with a positive pronumeral and a positive number instead of a negative pronumeral and a negative number, as in the case of the balance operation. Therefore, from this explanation, applying two inverse operations concurrently is likely to help learners better comprehend the solution procedure of two-step equations with a negative pronumeral.

One may argue that, overall, we could also apply two balance operations concurrently for each operational line. Nonetheless, applying two balance operations concurrently would incur twice the level of element interactivity as compared to applying two inverse operations concurrently. For example, with regard to 22 – 5*p* = 2, the two inverse operations involved are: (i) + 2 becomes– 2 and (ii) × (– 5) becomes ÷ 5. Applying these two inverse operations concurrently to solve for *p* would entail: 22–2 (left side) and 20 ÷ 5 (right side), which involves a total of six elements. In contrast, the two balance operations involved are: (i)– 22 on both sides and (ii) ÷ (– 5) on both sides. Applying these two balance operations concurrently to solve for *p* would entail:– 22 + 22, × (– 5) ÷ (– 5) (left side) and 2–22,– 20 ÷ (– 5) (right side), which has 12 elements in total. From this explanation, applying two balance operations concurrently not only requires learners to manipulate a negative pronumeral and a negative number, but it also incurs twice the level of element interactivity as compared to the application of two inverse operations concurrently. Consequently, applying two balance operations concurrently may overload the limited working memory and, on this basis, reduces effective learning.

### Conceptual knowledge and procedural knowledge

As noted previously, mathematics education researchers have argued that the balance method emphasizes the ‘ = ‘sign concept (e.g., +3 on both sides) [21], whereas the use of ‘change side, change sign’ within the inverse method lacks a mechanism to address the ‘ = ‘ sign concept [26]. Presumably, they concluded that the balance method can address the acquisition of both conceptual and procedural knowledge of linear equations, whereas the inverse method may only target the acquisition of procedure knowledge.

Research has indicated that both *conceptual knowledge* and *procedural knowledge* are essential components of personal competence in mathematics [30, 31]. Conceptual knowledge refers to a network of rich knowledge demonstrating the principle that underlines the relation between mathematical concepts [31]. Procedural knowledge refers to a sequence of steps involved to solve a problem. Connecting conceptual knowledge and procedural knowledge would facilitate more efficient application of mathematical procedures [32]. A review by Rittle-Johnson and her colleagues [33], interestingly, indicates a bidirectional relation between procedural knowledge and conceptual knowledge in mathematics learning. They attested to the gaining of procedural knowledge, consequently as a result of gaining conceptual knowledge and vice versa. For example, the acquisition of procedural knowledge for solving linear equations has been shown to enhance an understanding of the ‘ = ‘ sign concept in solving linear equations [34].

Fig 1 shows the interplay between one operational and two relational lines in the solution procedure, which in turn highlights the connection between conceptual knowledge and procedural knowledge in solving linear equations. For the balance method, performing– 4 on both sides of the equation so as to maintain its equality constitutes, in this case, procedural knowledge. For the inverse method, conceptualizing addition as inverse to subtraction, the learner moves + 4 from one side of the equation to become– 4 on the other side in order to preserve its equality. Such action constitutes procedural knowledge. The ability to judge the quantitative relationship thereby the right side of the equation equals to the left side with respect to the relational line constitutes conceptual knowledge (e.g., *x* + 3 = 15). Furthermore, if a learner could judge a pair of equations such as y + 4 = 1 and *y* + 4–4 = 1–4 or *y* + 4 = 1 and *y* = 1–4 as equivalent, this implies that the learner understands the ‘ = ‘ sign concept with respect to the operational line (i.e., conceptual knowledge). In the present study, we assessed student performance on both conceptual and procedural knowledge to validate the relative effectiveness of the balance method and the inverse method to acquire competence for linear equations. In line with our prior studies [e.g., 10], we used worked examples to facilitate learning to solve linear equations. We will review the use of worked examples as an instructional tool from a cognitive load perspective in the next section.

There is a volume of research that has focused on the significance of the *worked example effect*, which is one of the cognitive load effects [12, 35–38]. A worked example, in general, involves a problem and the provision of the solution steps and a final solution. The worked example effect occurs when exposure to worked examples enhances learning more than that of problem-solving without any guidance. Worked examples are particularly beneficial for novice learners who have limited prior knowledge in the domain of functioning [39]. Research has indicated the benefit of providing written explanation, which can serve as a prompt to facilitate learning from worked examples [40]. In relation to research on the worked example effect, researchers have advocated a sequence of studying a worked example paired with solving a similar problem [12]. Research has demostrated the worked example effect, for example, in the domain of algebra (e.g., *a* + *b*/*c* = *d*, solve for *b*) [36, 37]. We recently advanced this area of inquiry by focusing on the effectiveness of worked examples in facilitating learning to solve linear equations [9, 10, 29, 41] and percentage problems [42–44].

The worked example effect typically manifests in materials that are high rather than low in element interactivity [12]. In our prior studies, we observed an advantage of the inverse method over the balance method for linear equations that had high rather than low level of element interactivity [9, 10]. In a recent study, Chen, Retnowati, and Kalyuga [45] examined learner levels of expertise and varying levels of element interactivity associated with the solution steps of algebra expression problems (e.g., (2*x* – 5)(*x* + 1)). The worked example group outperformed the problem-solving group for the first solution step that had the highest level of element interactivity, demonstrating the worked example effect for novice learners.

### The present study: An experimental approach

*Instructional design* that considers the merit of cognitive load theory [12] is central to effective learning. One major issue, which we have studied, emphasizes the imposition of the level of element interactivity upon a particular instructional design [9, 10]. As noted previously, the balance method and inverse method differ in the operational line. Differential level of element interactivity arises from the interaction between elements that occurs on one side of the equation for the inverse method, but on both sides of the equation for the balance method. As a result, for each operational line, the ratio of the level of element interactivity between the balance method and the inverse method is 2:1. Moreover, the number of operational and relational lines in the solution procedure reflects the complexity of the equations, and therefore contributes to the level of element interactivity [28].

Advancing our previous research, we propose the undertaking of three comparative experiments that focus on differences pertaining to the balance method and the inverse method of learning for: (i) one-step equations (Experiment 1), (ii) two-step equations (Experiment 2), and (iii) one-step and two-step equations with a particular focus on equations with a negative pronumeral (Experiment 3) (Appendix A in S1 Appendix). The level of element interactivity depends on both the complexity of linear equations as well as the learner levels of expertise. In the present study, we examined the variable of the complexity of linear equations, which is influenced by the intrinsic nature of linear equations (e.g., a negative pronumeral), and the manner in which the linear equations are presented to learners (balance method *vs*. inverse method). Accordingly, we invited students who had no prior knowledge of linear equations to participate in the study.

Similar to our prior studies [9, 10, 29], we randomly assigned students who had no prior knowledge of solving linear equations to either the balance group or the inverse group. In order to investigate the progression of students’ learning of one-step and two-step equations via a specific method (balance or inverse), we allocated the same balance group (or inverse group) across the three experiments. We anticipated that students would gradually gain familiarity in using respective methods (i.e., balance or inverse) as they progressed through the three experiments.

We used the *pre-X-post* experimental design (*X* = intervention) for the three experiments to determine differences pertaining to the acquisition of conceptual knowledge and procedural knowledge on learning to solve linear equations. In line with the work of Rittle-Johnson and Star [46], we assessed procedural knowledge based on students’ accuracy in solving practice equations and post-test. We assessed conceptual knowledge somewhat differently from Johnson and Star [46], who instructed students to indicate whether the left side of the equation was equal to its right side (e.g., 2*x* – 5 + 91–91 = 2*x* – 5). Instead, we focused on two different tasks. The first task assessed students’ understanding of the ‘ = ‘ sign concept with respect to the relational line (e.g., *x* + 6 = 11). The second task evaluated students’ understanding of the ‘ = ‘ sign concept with respect to the operational line. Essentially, this requires students to judge whether a pair of equations are equivalent after an operation has been performed on the equation, irrespective of the method used (e.g., *x* + 4 = 6 and *x* + 4–4 = 6–4, balance operation; *x* + 4 = 6 and *x* = 6–4, inverse operation). It should be noted that the use of multiple tasks to assess conceptual knowledge increases the reliability of assessing the same concept [47]. For example, it is advisable to use both order-irrelevance and cardinality to assess the key concept of *counting*.

In our prior work [9], the application of inverse operation only occurred in a sequential manner for the operational line. In this study, we investigated the application of inverse operation sequentially (Experiments 1 and 2) as well as concurrently (Experiment 3) for the operational line (Appendix A in S1 Appendix). Regarding linear equations with a negative pronumeral (e.g., one-step equations), performing two inverse operations concurrently for the operational line will incur fewer operational and relational lines than performing two balance operations sequentially across two operational lines. Therefore, we predicted that the inverse method would impose lower cognitive load than the balance method due to fewer operational and relational lines.

Differential level of element interactivity between the balance operation and the inverse operation is of primary interest in the present study. Therefore, we also measured the mental effort invested in judging pairs of equations presented in both the balance operation (e.g., *x* + 3 = 9 and *x* + 3–3 = 9–3) and the inverse operation (e.g., *x* + 3 = 9 and *x* = 9–3). Our aim was to validate the claim in prior studies [e.g., 9] that the inverse operation imposes lower cognitive load than the balance operation.

## Experiment 1: One-step equations

We compared the balance method and the inverse method on learning one-step equations that have one operational and two relational lines (e.g., *n* + 4 = 13), and two operational and three relational lines (e.g., 15 –*a* = 10). Differential number of operational and relational lines corresponds to differential level of element interactivity (low *vs*. high). We used a 2 (method: balance *vs*. inverse) × 2 (type of equation: low element interactivity *vs*. high element interactivity) factorial design to assess the acquisition of procedural knowledge of one-step equations. The first independent variable (method) was a between-subjects factor, and the second independent variable (type of equation) was a within-subjects repeated measures factor. The dependant variables were scores on the practice equations and a post-test.

Assessment of conceptual knowledge was based on students’ understanding of the ‘ = ‘ sign concept with respect to the relational line and operational line in a concept test. A Chi-square test was used to assess the acquisition of conceptual knowledge between the balance method and the inverse method with respect to the relational line. We used 2 (method: balance *vs*. inverse) × 2 (concept: balance operation *vs*. inverse operation) factorial design to assess the acquisition of conceptual knowledge with respect to the operational line. The first independent variable (method) was a between-subjects factor, and the second independent variable (concept) was a within-subjects repeated measures factor. The dependant variables were scores on the balance operation and the inverse operation.

We proposed a few research questions to examine the level of element interactivity and instructional design, particularly for linear equations that have a negative pronumeral. Our research questions include the following: (i) will both the balance method and the inverse method perform better on procedural knowledge for low rather than high element interactivity equations?, (ii) is there a differential performance on procedural knowledge favouring the inverse method for high element interactivity equations?, and (iii) is there a difference between the balance method and the inverse method in regard to an understanding of the ‘ = ‘ sign concept with respect to the relational line and the operational line? To answer the three research questions, we formulated two hypotheses for testing:

*Hypothesis 1*:
  - The acquisition of procedural knowledge would favour low element interactivity equations regardless of the balance method or the inverse method.
  - The acquisition of procedural knowledge on high element interactivity equations would favour the inverse method.
*Hypothesis 2*:
  - The level of element interactivity of the relational line is determined by its intrinsic nature. Hence, the balance method and the inverse method would not expect to exhibit differential performance on the concept test with respect to the relational line.
  - The inverse operation imposes half the number of interactive elements as the balance operation on each operational line. Thus, performance on the concept test with respect to the operational line would favour the inverse operation, regardless of the balance method or the inverse method.

### Method

#### Participants

We invited 75 eighth-grade Chinese students (females = 48, males = 27) whose mean age was 14.12 (SD = 0.16) to participate in the study. We obtained ethics clearance from the Research Ethics Committee, University of New England (Approval Number: HE13–262) prior to data collection. Student who consented to participate in the study were from two classes of a school in an Asian country. They followed the National Curriculum for Mathematics Education. The medium of instruction for science and mathematics subjects was English language. The ethnic distribution of the students was Foochow (85%) and Hokkien (15%), respectively. Students had learned algebra expression problems (e.g., 3*a* + 9*b* –*a*) but they had not learned how to solve linear equations. We obtained ethics approval prior to data collection. The experiment was conducted at the beginning of the second school term during which the topic of linear equations was scheduled for coverage.

#### Materials

The materials for this experiment were adapted from a previous study [29] and consisted of the following: (1) an instruction sheet, (2) 12 pairs of acquisition equations (Appendix B in S1 Appendix), (3) a pre-test and a post-test that shared similar content (Appendix C in S1 Appendix), and (4) a concept test (Appendix D in S1 Appendix).

*Instruction sheet*. The instruction sheet presented the definition of an equation, the solution steps for solving linear equations, the scaffold of the balance operation and the inverse operation, and four worked examples (Appendix B in S1 Appendix). The *balance scale* was used to illustrate the concept of balance operation (Smith et al., 2011). The inverse operation highlights the inverse relationship between mathematical operations (e.g., 5 + 2 = 7 is the same as 5 = 7–2). Research has designed prompting in the form of written explanation to foster learning from worked examples [40]. Accordingly, we provided ‘prompting’ for both the balance method and the inverse method to emphasize the interplay between procedural knowledge and conceptual knowledge in solving linear equations. For the balance method, each worked example was aided by a prompt showing “– 2 on both sides”. For the inverse method, each worked example was aided not only by a prompt “+ 2 becomes– 2”, but also an arrow to indicate performance of an inverse operation. We postulated that prompting would assist students to understand the application of mathematical operations in solving linear equations.

*Pairs of acquisition equations*. The acquisition equations resembled the test equations in the post-test. Each pair of the acquisition equations (balance or inverse) comprised a worked example, paired with a practice equation, which shared a similar problem structure [48]. In line with previous worked examples research, students were instructed to study a worked example alternated with solving a practice equation [36, 37]. Acquisition of procedural knowledge and conceptual knowledge of linear equations would expect to occur after students had completed multiple example-equation pairs. Nonetheless, they could also benefit from studying the materials in the instruction sheet. Of those 12 pairs of acquisition equations, eight had one operational and two relational lines (low element interactivity), and four had two operational and three relational lines (high element interactivity). Across the multiple pairs of acquisition equations, we graded the practice equations but not the worked examples.

*Pre-test and post-test*. Both the pre-test and the post-test had similar content (see Appendix C in S1 Appendix), which consisted of 30 one-step equations [49]. The 30 one-step equations ranged in complexity (e.g., *a* + 8 = 17, 1 = 2*n*, etc.), and of these, 24 had one operational and two relational lines, and six had two operational and three relational lines. The pre-test was used as a basis to establish group equivalency prior to intervention.

*Concept test*. The concept test evaluated students’ understanding of the ‘ = ‘ sign concept in relation to both relational and operational lines. We assessed their understanding of the role of ‘ = ‘ sign in the relational line, *x* + 6 = 11. The concept test with respect to the operational line consisted of eight pairs of equations (e.g., *x* + 3 = 5 and *x* + 3–3 = 5–3, balance operation; and *x* + 3 = 5 and *x* = 5–3, inverse operation, Appendix D in S1 Appendix). For an equal distribution, we allocated four pairs of equations that showed the balance operation, and four pairs of equations that showed the inverse operation. Students’ task (i.e., balance group or inverse group) was to judge whether each pair of equations was equivalent.

#### Procedure

**Fig 2:**
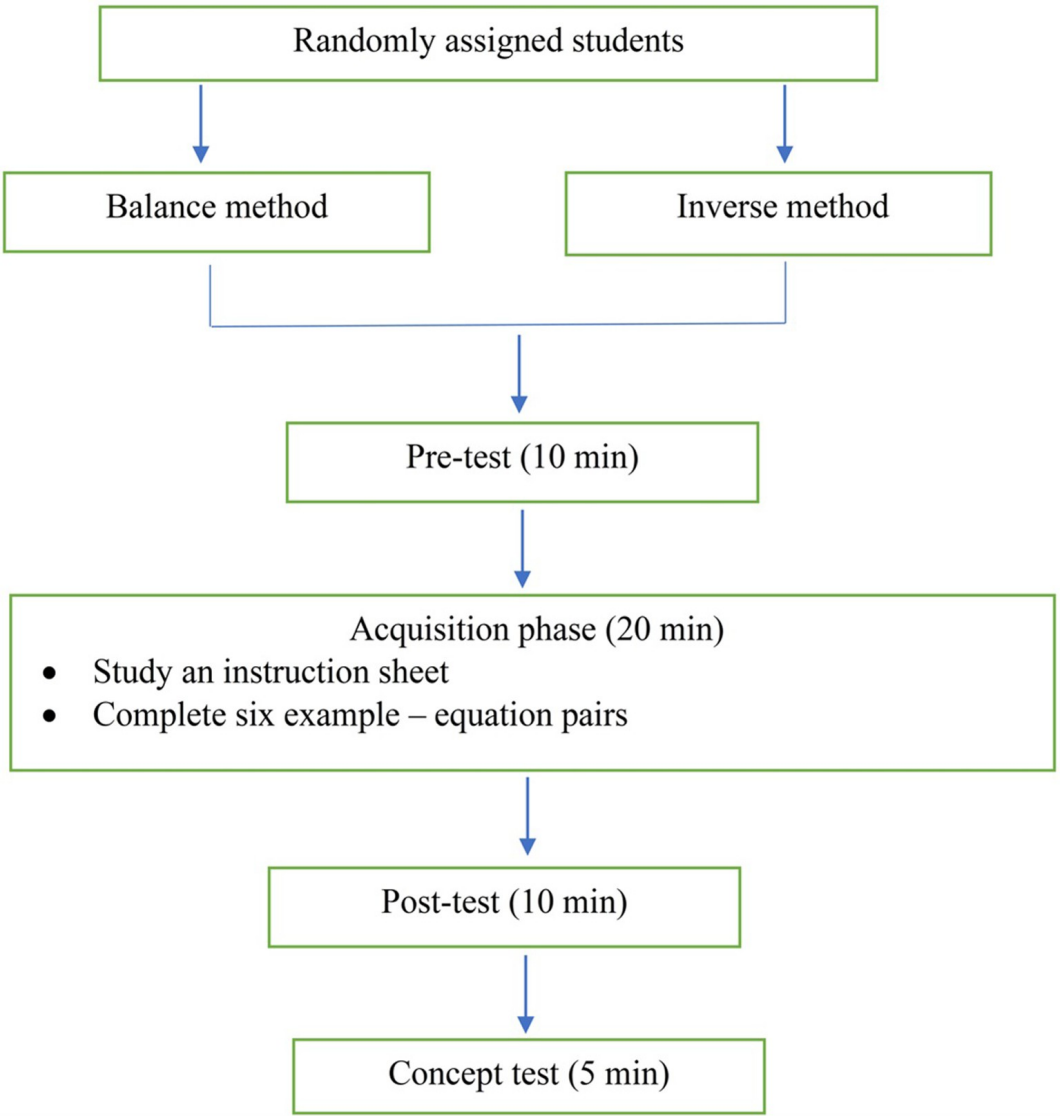
General procedure across three experiments. Day 1: Experiment 1 (one-step equations). Day 3: Experiment 2 (two-step equations). Day 8: Experiment 3 (one-step and two-step equations). The concept test comprised three questions (10 min). Applied the inverse operation concurrently.

The flow-chart in Fig 2 shows the general procedure across Experiments 1, 2 and 3, which is similar to our previous undertakings [10]. Grouping testing was administrated to students under supervision. In Experiment 1, seventy-five students were randomly allocated to the balance group (N = 37 students) or the inverse group (N = 38 students). The instruction reflected the following:

- The teacher informed the students that they were going to learn how to solve linear equations.
- The teacher asked the students to complete a series of written tasks, which consisted of a pre-test (10 min), an acquisition phase (an instruction sheet + acquisition equations) (20 min), a post-test (10 min), and a concept test (5 min).

Students were provided with written instruction and asked to work through the tasks, individually, for each phase. They were allowed to seek help during the acquisition phase, but not test phases (pre-test, post-test and concept test). First, all students completed a pre-test (5 min). Having collected the pre-test, the teacher then distributed an instruction sheet (balance or inverse) to each student. Students were told to study the instruction sheet (5 min). Once the 5 min had expired, the teacher distributed a booklet that comprised 12 pairs of acquisition equations (inverse or balance) to each student. Students were instructed to study a worked example paired with solving a practice equation that shared a similar problem structure. Students could consult the instruction sheet and seek help if they could not understand the worked examples. They did not receive assistance when solving the practice equations. They were asked to review their work if they finished earlier than the allocated time. During the acquisition phase, the balance group or the inverse group studied an instruction sheet that displayed four worked examples and completed 12 pairs of acquisition equations (24 acquisition equations). Once the teacher had collected the instruction sheet and the acquisition equations, all students undertook a post-test (10 min). Lastly, upon the completion of the post-test, students then completed a concept test (5 min).

### Scoring and coding

For the pre-test, the practice equations and the post-test, we allocated one point for a correct answer, with or without the inclusion of solution steps. Because we wanted to assess students’ procedural knowledge in solving linear equations, we marked the solution as correct if students could perform an operation (balance or inverse) accurately but made computational errors (e.g.,– 3 + 7 = – 4). However, students would receive a zero point, though, if they made a procedural mistake in performing an operation. For example, students wrote + 7 on both sides instead of– 7 on both sides when solving the equation of, say, 7 –*n* = 0. We used mean proportion scores owing to the unequal number of the two types of equations across the pre-test, the practice equations, and the post-test.

The first question in the concept test assessed the ‘ = ‘ sign concept with respect to the relational line. In their study, Asquith, Stephens, Knuth, and Alibali [50] regarded an answer such as “the same as” as a reflection of students’ understanding of the ‘ = ‘ sign concept. Accordingly, we assigned one point if students provided an answer such as “equal to, the same as”. However, students received a zero point if they omitted an answer or that their answer was obscure. Regarding the second question, we assigned one point if students could accurately judge a pair of equations as being equivalent. A researcher and a research assistant scored the pre-test, the practice equations, the post-test, and the concept test. Differences in scores between them were resolved through discussion. Overall, the inter-scorer agreement was above .90, indicating that both scorers scored similarity for over 90% of the items across the practice equations, the post-test, and the concept test. This manner of achieving inter-scorer reliability was used for all three experiments in the study. The Cronbach’s alpha values for the pre-test, the practice equation, and the post-test were .87, .79, and .90, respectively, suggesting that the items within each test have relatively high internal consistency [51].

In relation to data analysis, we conducted a t-test to assess prior knowledge of both methods. To test hypothesis 1, we performed 2 × (method: balance *vs*. inverse) × 2 (type of equation: low element interactivity *vs*. high element interactivity) ANOVA on practice equations and the post-test. To test hypothesis 2, we conducted a Chi-square test for the concept test with respect to the relational line, and we performed 2 × (method: balance *vs*. inverse) × 2 (concept: balance operation *vs*. inverse operation) ANOVA for the concept test with respect to the operational line.

### Results and discussion

**Fig 3:**
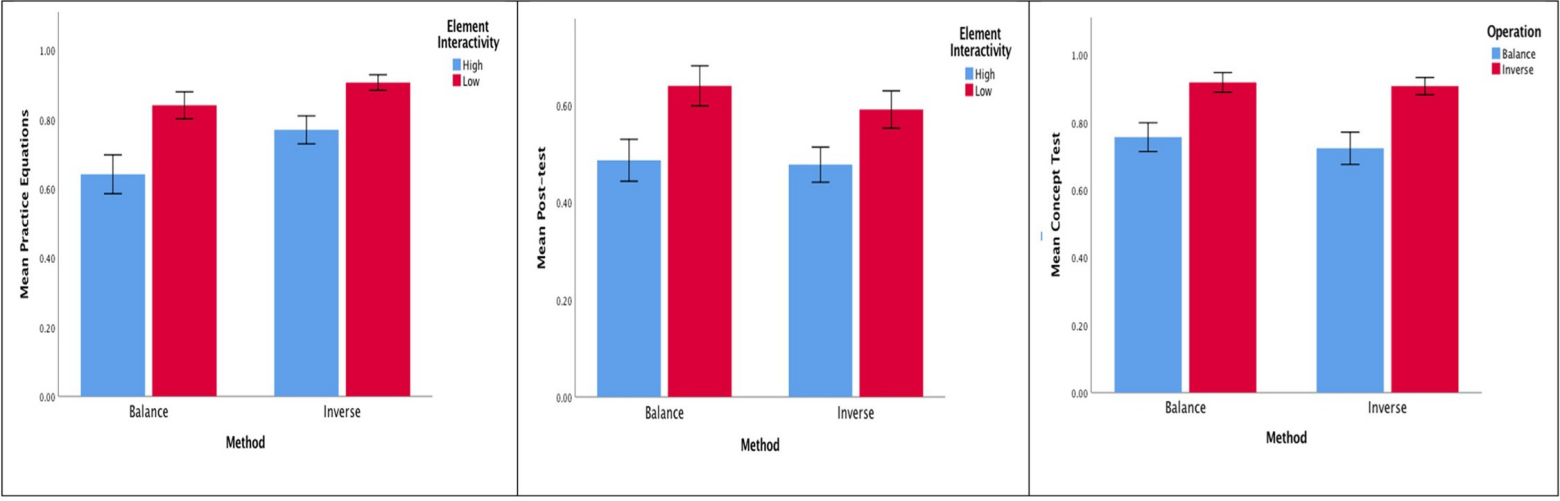
For One-step Equations in Experiment 1, the Effects of Method (balance vs. inverse) on: (a) Practice Equations, (b) Post-test, and (c) Concept Test. Error Bars are Standard Errors.

The means and *SD*s of the pre-test, the practice equations, the post-test, and the concept test for one-step equations are shown in Table 1. The means and standard errors of the practice equations, the post-test, and the concept test are displayed in Fig 3. Students used trial and error method to solve the pre-test. The two groups did not differ for the pre-test, indicating no difference between the two groups before the intervention, *t*(73) = 1.49, *SE* = 0.05, *p* = 0.14.

**Table 1:** Performance outcomes of pre-test. Practice Equations, Post-test, and Concept Test for One-Step and Two-Step Equations in Experiments 1 and 2.

	Balance Method		Inverse Method	
	*M*	*(SD)*	*M*	*(SD)*
Experiment 1	*n* = 37		*n* = 38	
One-step equations (proportion)				
Pre-test	0.50	(0.21)	0.43	(0.19)
Practice equations				
One operational + two relational lines	0.84	(0.24)	0.92	(0.12)*
Two operational + three relational lines	0.64	(0.34)	0.78	(0.25)*
Post-test				
One operational + two relational lines	0.64	(0.25)	0.59	(0.24)
Two operational + three relational lines	0.49	(0.26)	0.48	(0.22)
Concept test (proportion)				
Balance operation	0.76	(0.26)	0.72	(0.30)
Inverse operation	0.92	(0.18)	0.91	(0.16)
Experiment 2	*n* = 36		*n* = 38	
Two-step equations (proportion)				
Pre-test	0.29	(0.30)	0.32	(0.27)
Practice equations				
Two operational + three relational lines	0.82	(0.19)	0.81	(0.12)
Two operational + four relational lines	0.72	(0.28)	0.75	(0.30)
Post-test				
Two operational + three relational lines	0.41	(0.28)	0.44	(0.30)
Two operational + four relational lines	0.46	(0.34)	0.46	(0.32)
Concept test (proportion)				
Balance operation	0.71	(0.39)	0.61	(0.41)
Inverse operation	0.81	(0.28)	0.85	(0.25)

*Note*: One-step equations: 12 practice equations, pre-test was identical to post-test (30 equations), balance operation (4 pairs of equations), and inverse operation (4 pairs of equations). Two-step equations: 10 practice equations, pre-test was identical to post-test (30 equations), balance operation (3 pairs of equations), and inverse operation (3 pairs of equations). *P < 0.05.

For the practice equations, a significant effect on the type of equation was found, *F*(1, 73) = 29.63, *p* < .001, partial η^2^ = 0.29, indicating that both groups scored a higher mean proportion on low (0.84 *vs*. 0.92) rather than high element interactivity equations (0.64 *vs*. 0.78). The interaction between the method × practice equation was not significant, *F*(1, 73) = 0.86, *p* = 0.36, partial η^2^ = 0.01. However, the method effect was significant, *F*(1, 73) = 4.81, *p* = 0.03, partial η^2^ = 0.06, indicating that the inverse group outperformed the balance group.

For the post-test, once again, a significant effect on the type of equation was observed, *F*(1, 73) = 28.30, *p* < .001, partial η^2^ = 0.28, indicating that both groups scored a higher mean proportion on low (0.64 *vs*. 0.59) rather than high element interactivity equations (0.49 *vs*. 0.48). Neither the method × type of equation interaction effect, *F*(1, 73) = 0.57, *p* = 0.45, partial η^2^ = 0.01, nor the method effect was significant, *F*(1, 73) = 0.30, *p* = 0.59, partial η^2^ = 0.00.

Concerning the concept test, the first question assessed an understanding of the ‘ = ‘ sign concept with respect to the relational line. The balance group and the inverse group scored 57% and 66%, respectively. A Chi-square test showed no difference between the two groups, χ^2^ (1, N = 75) = 0.65, *p* = 0.42. The second question assessed students’ understanding of the ‘ = ‘ sign concept with respect to the operational line. A 2 (method) × 2 (concept) ANOVA indicated neither the method × concept interaction effect, *F*(1, 73) = 0.10, *p* = 0.75, *η*^*2*^ = 0.00, nor the method effect, *F*(1, 73) = 0.31, *p* = 0.58, *η*^*2*^ = 0.00. In contrast, a significant concept effect was observed, *F*(1, 73) = 24.37, *p* < .001, *η*^*2*^ = 0.25, indicating that both groups scored a higher mean proportion on the inverse operation (0.92 *vs*. 091) than the balance operation (0.76 *vs*. 0.72).

Overall, the results established from Experiment 1 supported most of the proposed hypotheses. As hypothesized, the acquisition of procedural knowledge was more pronounced for the practice questions and post-test, which involved low element interactivity knowledge (one operational line and two relational lines), irrespective of the balance group or the inverse group. The balance group was inferior to the inverse group on practice equations, but not the post-test. Such results partially support hypothesis 1 (ii). As hypothesized, regarding the concept test, the inverse group was on par with the balance group with respect to the relational line, and both groups performed better on the inverse operation than the balance operation with respect to the operational line.

## Experiment 2: Two-step equations

The objective of Experiment 2 was to extend the findings of Experiment 1 by using two-step equations, which would impose higher element interactivity than one-step equations. The two-step equations comprised two operational and three relational lines (e.g., 3*a* + 2 = 8), and two operational and four relational lines (e.g., $\frac{\left(5+a\right)}{2}$ = 4). Thus, the two-step equations exhibited two levels of element interactivity because of a difference of one relational line. Similar to Experiment 1, we conducted a 2 (method) × 2 (type of equation) and 2 (method) × (concept) factorial design to assess students’ acquisition of procedural knowledge and conceptual knowledge on learning two-step equations. We tested two hypotheses that were similar to Experiment 1.

### Method

#### Participants

This experiment was conducted two days after Experiment 1. Students who were allocated to the balance group in Experiment 1 remained in the balance group (*N* = 36). One student in the balance group was absent, resulting in 36 instead of 37 students. Likewise, students who were allocated to the inverse group in Experiment 1 remained in the inverse group (*N* = 38).

#### Materials and procedure

The procedure was similar to Experiment 1 (Fig 2). There were four sets of materials: (1) an instruction sheet, (2) 10 pairs of acquisition equations, (3) a pre-test that shared similar content to a post-test (Appendix C in S1 Appendix), and (4) a concept test (Appendix D in S1 Appendix). The layout of the instruction sheet was the same as in Experiment 1 but the content was two-step equations. Regarding the acquisition equations, 8 pairs had two operational and three relational lines (low element interactivity), and two pairs had two operational and four relational lines (high element interactivity).

The post-test consisted of 30 two-step equations, which ranged in complexity (e.g., 2*b* – 11 = – 3, $\frac{a}{3}$– 4 = 2, etc.). Of these 30 two-step equations, 25 had two operational and three relational lines, and five had two operational and four relational lines (Appendix C in S1 Appendix).

Similar to Experiment 1, the two questions in the concept test assessed students’ understanding of the ‘ = ‘ sign concept with respect to the relational and operational lines (Appendix D in S1 Appendix). Half of the six pairs of equations were presented via the balance operation, and the other half was presented via the inverse operation.

### Scoring and coding

Both scoring and coding were the same as in Experiment 1. The inter-scorer agreement was above .90 for the pre-test, the practice equations, the post-test and the concept test. The Cronbach’s alpha for the practice equations was 0.41 after deleting the 10^th^ item. This rather low Cronbach’s alpha value may be due to a small number of practice equations coupled with a range of two-step equations. The pre-test and the post-test had the same Cronbach’s alpha of .94. The data analysis was similar to Experiment 1.

### Results and discussion

**Fig 4:**
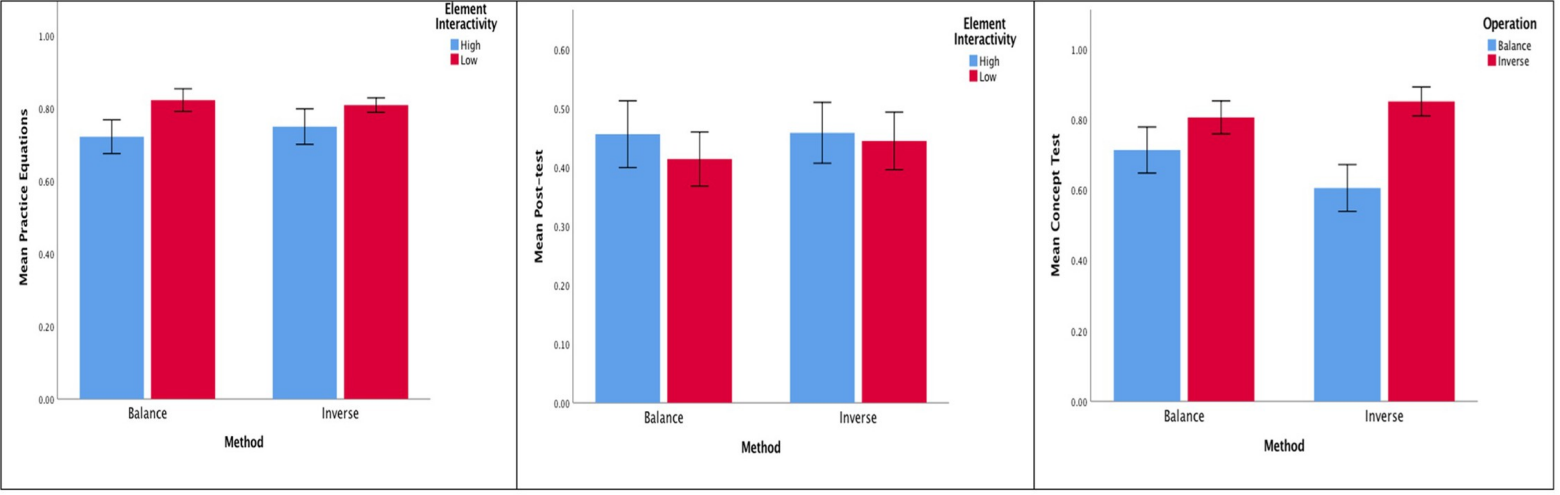
For Two-step Equations in Experiment 2, the Effects of Method (balance vs. inverse) on: (a) Practice Equations, (b) Post-test, and (c) Concept Test. Error Bars are Standard Errors.

Table 1 shows the means and *SD*s of the pre-test, the practice equations, the post-test, and the concept test for the two-step equations. Fig 4 shows the means and standard errors of the practice equations, the post-test, and the concept test. No difference between the two groups was found for the pre-test, *t*(72) = 0.00, *SE* = 0.06, *p* = 1.00, indicating group equivalence in terms of students’ ability to solve two-step equations before the intervention. For the practice equations, a significant effect on the type of equation was obtained, *F*(1, 72) = 4.66, *p* = 0.03, partial η^2^ = 0.06, suggesting that both groups scored a higher mean proportion on low (0.82 *vs*. 0.81) rather than high element interactivity equations (0.72 *vs*. 0.75). The method × type of equation interaction was not significant, *F*(1, 72) = 0.31, *p* = 0.58, partial η^2^ = 0.00. Likewise, the method effect was not significant, *F*(1, 72) = 0.03, *p* = 0.86, partial η^2^ = 0.00. Apart from the method effect, other results are similar to Experiment 1.

For the post-test, a nonsignficant effect on the type of equation was observed, *F*(1, 72) = 1.61, *p* = 0.21, partial η^2^ = 0.02, nor a significant effect on the interaction between the method × type of equation, *F*(1, 72) = 0.42, *p* = 0.52, partial η^2^ = 0.01, or the method effect, *F*(1, 72) = 0.06, *p* = 0.81, partial η^2^ = 0.00. Such results are similar to Experiment 1.

Concerning students’ understanding of the ‘ = ‘ sign concept with respect to the relational line, the balance group and inverse group scored 67% and 53%, respectively. No difference between the two groups was found, χ^2^ (1, N = 74) = 1.51, *p* = 0.22. Regarding students’ understanding of the ‘ = ‘ sign with respect to the operational line, a 2 (method) × 2 (concept) ANOVA revealed neither the method × concept effect, *F*(1, 72) = 3.10, *p* = 0.08, partial η^2^ = 0.04, nor the method effect, *F*(1, 72) = 0.22, *p* = 0.64, partial η^2^ = 0.00, was significant. Such results are similar to Experiment 1. However, consistent with Experiment 1, a significant concept effect was found, *F*(1, 72) = 15.13, *p* < .001, partial η^2^ = 0.17, indicating that both groups scored a higher mean proportion on the inverse operation (0.81 *vs*. 0.85) than the balance operation (0.71 *vs*. 0.61).

The pattern of results coincided with some of the results established in Experiment 1. The acquisition of the procedural knowledge was limited to the practice equations whereby both groups performed better on the two-step equations, which involved low element interactivity knowledge. Such results partially support hypothesis 1 (i). As hypothesized, for the concept test, similar to Experiment 1, both groups did not differ with respect to the relational line and both groups performed better on the inverse operation than the balance operation with respect to the operational line.

On the whole, the results do not support the majority of the hypotheses. It is plausible to suggest that a difference of one extra relational line between the two types of two-step equations (3 *vs*. 4) was not substantial enough to result in differential learning outcomes. It appears that the inverse method was not better than the balance method for two-step equations with a negative pronumeral–especially when these were presented using two inverse operations sequentially, which resulted in sharing a similar number of operational and relational lines as those found in the balance method (Appendix A in S1 Appendix).

## Experiment 3: One-step and two-step equations

As shown in Experiment 2, the inverse method was no better than the balance method for learning two-step equations. Presumably, the gap between the two levels of element interactivity within the two-step equations was not large enough to result in differential learning outcomes. To counter this contention, we designed Experiment 3 with a focus on a bigger difference between the two levels of element interactivity within one-step and two-step equations. So, overall, Experiment 3 built on the two previous experiments by investigating the effect of the balance method and the inverse method on one-step and two-step equations that have a positive pronumeral (e.g., 3*x* – 7 = 22) and a negative pronumeral (e.g., 8 – 4*x* = 16). It should be noted that the point of reference for comparison between the balance method and the inverse method is still based on the number of operational and relational lines. As noted earlier, similar to negative number, the concept of negative pronumeral poses great difficulty for students. In particular, for the balance method, the presence of a negative pronumeral increases the number of operational and relational lines (e.g., 11– *x* = 7, Appendix A in S1 Appendix).

In the previous two experiments, the one-step equations or two-step equations with a positive or a negative pronumeral were mixed in one experiment. In contrast, for this experiment, we divided one-step and two-step equations into two components: (1) one-step and two-step equations with a positive pronumeral (low element interactivity), and (2) one-step and two-step equations with a negative pronumeral (high element interactivity). For the inverse method, we applied two inverse operations concurrently instead of sequentially as in the case of Experiments 1 and 2, resulting in fewer operational and relational lines (Appendix A in S1 Appendix).

We proposed three hypotheses, two of which (Hypotheses 1 and 2) were similar to Experiment 1. The inverse method imposes half the number of the interactive elements as the balance method. Thus, the mental effort invested to process the balance operation and the inverse operation is of particular interest. We postulated that differential mental effort invested to process the balance operation and the inverse operation would reflect differential level of element interactivity for the balance operation and the inverse operation. We hypothesized the following:

*Hypothesis 3*:
  - Regardless of the balance group or the inverse group, students would invest a higher mental effort in judging the balance operation than the inverse operation.

### Method

#### Participants

This experiment occurred four days after Experiment 2. Participants in this experiment were the same students in Experiments 1 and 2. Again, the balance group remained as the balance group, whereas the inverse group remained as the inverse group. On the day of data collection, two students from each group were absent and three students did not complete all phases of the experiment (two from the balance group and one from the inverse group). Consequently, there were 33 students in the balance group and 35 students in the inverse group in the final analysis.

#### Materials and procedure

The procedure was similar to Experiment 1 or Experiment 2 (Fig 2). The materials comprised: (1) an instruction sheet, (2) 12 pairs of acquisition equations, (3) a pre-test that shared similar content to the post-test (Appendix C in S1 Appendix), and (4) a concept test (Appendix D in S1 Appendix). The layout of the instruction sheet was the same as in Experiment 1 or Experiment 2 but the content was one-step and two-step equations. The worked examples of the inverse method illustrated the use of two inverse operations concurrently to solve linear equations with a negative pronumeral. The first half of the acquisition equations involved a positive pronumeral, and the second half involved a negative pronumeral. There were two types of one-step and two-step equations with a positive pronumeral: (1) one operational line and two relational lines (e.g., *x*– 4 = 9), and (2) two operational and three relational lines (e.g., 2*x* + 11 = 33). Therefore, there were two levels of element interactivity for one-step and two-step equations with a positive pronumeral, owing to the presence of varying number of operational and relational lines.

There were two types of one-step and two-step equations with a negative pronumeral, whereby the coefficient of the negative pronumeral is either one (e.g., 11 –*x* = 7) or more than one (e.g., 5 – 3*t* = –19). When the coefficient of the negative pronumeral is one (e.g., 11 –*x* = 7), the method of learning (balance or inverse) determines the number of the operational and relational lines. As shown in Appendix A in S1 Appendix, the balance method has more operational lines (2 *vs*. 1) and relational lines (3 *vs*. 2) than the inverse method. This is due to the flexibility of the inverse operation that allows the application of two inverse operations concurrently in one operational line. In contrast, though, when the coefficient of the negative pronumeral is more than one (e.g., 5 – 3*t* = –19), then the solution procedure of both the balance and inverse methods would involve two operational and three relational lines, regardless of whether we apply the inverse operation sequentially or concurrently. Thus, overall, one-step and two-step equations with a negative pronumeral exhibit differential level of element interactivity due to the presence of varying number of operational and relational lines.

Of those 16 one-step and two-step equations in the post-test, nine had a positive pronumeral, and seven had a negative pronumeral. The level of element interactivity, and thus, the number of operational and relational lines involved in the post-test, was similar to the practice equations.

The concept test comprised three questions. The first two questions were similar to Experiments 1 and 2. The third question comprised two equations such as *x–* 2 = 10 and 7 –*y* = –1. Each equation was presented in both the balance method and the inverse method in relation to the operational line (e.g., *x*– 2 = 10 and *x*– 2 + 2 = 10 + 2, balance operation; *x*– 2 = 10, *x* = 10 + 2, inverse operation), resulting in a total of four pairs of equations. The main task, in this case, was for students to: (i) judge whether a pair of equations was equivalent in relation to the ‘ = ‘ sign concept of the operational line, and (ii) indicate their mental effort invested in judging the pair of equations on a Likert scale that ranged from *extremely low mental effort (1) to extremely high mental effort (9*) [52]. A particular rating (response) of the item would, in this case, reflect and indicate a student’s level of mental effort that has been invested in judging the operation (balance or inverse) [53].

The procedure was similar to Experiments 1 and 2 with one minor difference in the concept test (see Fig 2). Students completed three questions in the concept test which took 10 min.

### Scoring and coding

Both scoring and coding were the same as in Experiments 1 and 2. The inter-scorer agreement was above .90 for the pre-test, the practice equations, the post-test and the concept test. The Cronbach’s alpha for the pre-test, the practice equations and the post-test were .81, .80, and .72, respectively. To test the hypotheses 1 and 2, we analysed the data similar to Experiment 1 or Experiment 2. In addition, to test hypothesis 3, we performed a 2 (method) × 2 (concept) ANOVA on mental effort.

### Results and discussion

**Fig 5:**
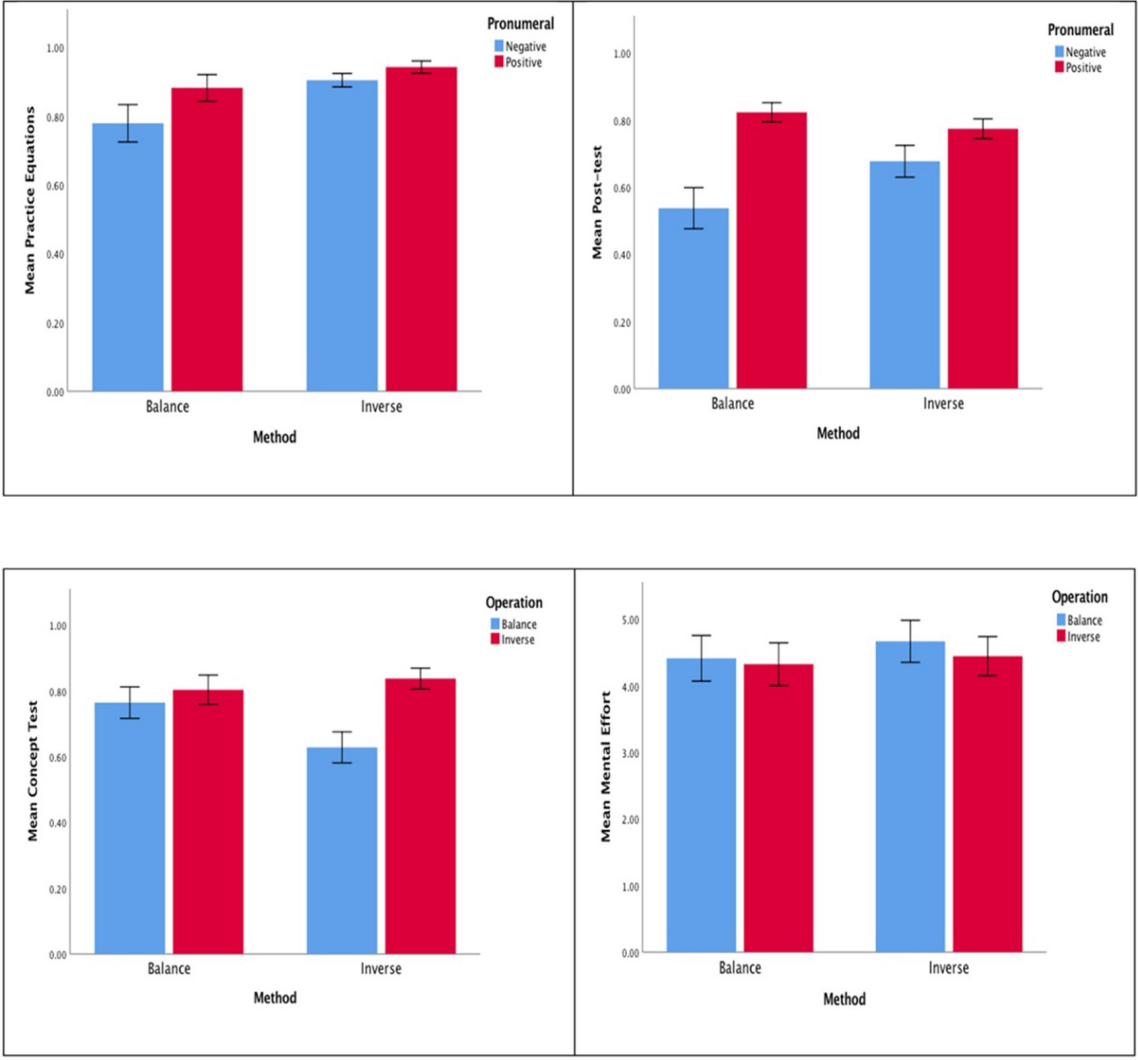
For One-step and Two-step Equations with a Positive and a Negative Pronumeral in Experiment 3, the Effects of Method (balance vs. inverse) on: (a) Practice Equations, (b) Post-test, (c) Concept Test, and (d) Mental Effort. Error Bars are Standard Errors.

Table 2 shows the means and *SD*s of the pre-test, the practice equations, the post-test, and the concept test, and indication of mental effort for the balance operation and inverse operation. Fig 5 displays the means and standard errors of the practice equations, the post-test, the concept test, and the mental effort. There was no difference between the two groups for the pre-test, *t*(66) = 0.34, *SE* = 0.06, *p* = 0.74, indicating group equivalence in solving one-step and two-step equations before the intervention.

**Table 2:** Performance outcomes of pre-test, practice equations, post-test, concept test and mental effort for equations with a positive and a negative pronumeral in Experiment 3.

	Balance Method *n* = 33		Inverse Method *n* = 35	
	*M*	*(SD)*	*M*	*(SD)*
One-step and two-step equations (proportion)				
Pre-test	0.64	(0.22)	0.62	(0.24)
Equation with a positive pronumeral				
Practice equations	0.88	(0.23)	0.94	(0.11)*
Post-test	0.82	(0.17)	0.77	(0.17)
Equation with a negative pronumeral				
Practice equations	0.77	(0.32)	0.90	(0.12)*
Post-test	0.53	(0.35)	0.68	(0.27)*
Concept test (proportion)				
Balance operation	0.76	(0.28)	0.63	(0.27)
Inverse operation	0.81	(0.26)	0.83	(0.20)
Mental effort				
Balance operation	4.41	(1.88)	4.70	(1.86)
Inverse operation	4.33	(1.91)	4.47	(1.87)

Note: Equation with a positive pronumeral: one operational + two relational lines and two operational + three operational lines, practice equations (6 equations), pre-test was identical to post-test (8 equations). Equation with a negative pronumeral: two operational + three relational lines, practice equations (6 equations), pre-test is identical to post-test (8 equations). Concept test: Balance operation (5 pairs of equations), and inverse operation (3 pairs of equations). Mental effort: Balance operation (2 pairs of equations), and inverse operation (2 pairs of equations). *P < 0.05.

Regarding the practice equations, a significant effect on the type of equation was foound, *F*(1, 66) = 7.52, *p* = 0.01, partial η^2^ = 0.10, suggesting that both groups scored a higher mean proportion on equations with a positive pronumeral (0.88 *vs*. 0.94) than equations with a negative pronumeral (0.77 *vs*. 0.90). These results are similar to those results obtained in Experiments 1 and 2. The method × practice equation interaction was not significant, *F*(1, 66) = 1.80, *p* = 0.18, partial η^2^ = 0.03. However, the method effect was significant, *F*(1, 66) = 4.93, *p* = 0.03, partial η^2^ = 0.07, revealing that the inverse group outperformed the balance group.

Again, for the post-test, a significant effect on type of equation was observed, *F*(1, 66) = 23.93, *p* < .001, partial η^2^ = 0.27, indicating that both groups scored a higher mean proportion on equations with a positive pronumeral (0.82 *vs*. 0.77) than equations with a negative pronumeral (0.53 *vs*. 0.68). The method × type of equation interaction was significant, *F*(1, 66) = 6.65, *p* = 0.01, partial η^2^ = 0.09 but not the method effect, *F*(1, 66) = 1.28, *p* = 0.26, partial η^2^ = 0.02. Simple effects tests revealed that the inverse group outperformed the balance group for equations with a negative pronumeral, *F*(1, 66) = 4.06, *p* = 0.05, partial η^2^ = 0.06 but not equations with a positive pronumeral, *F*(1, 66) = 1.31, *p* = 0.26, partial η^2^ = 0.02.

For the first question in the concept test, which was related to the role of the ‘ = ‘ sign concept with respect to the relational line of the equation, the balance and inverse groups scored 49% and 57%, respectively. No difference between the two groups was observed, χ^2^(1, N = 68) = 0.51, *p* = 0.48. The results are consistent with those results found in Experiments 1 and 2.

For the second question, which was related to the role of the ‘ = ‘ sign with respect to the operational line, a 2 (method) × 2 (concept) ANOVA yielded a significant concept effect, *F*(1, 66) = 13.35, *p* < .001, *η*^*2*^ = 0.17, indicating that both groups scored a higher mean proportion on the inverse concept (0.81 *vs*. 0.83) than the balance concept (0.76 *vs*. 0.63). Such results again are similar to those obtained in Experiments 1 and 2. The method × concept interaction was significant, *F*(1, 66) = 4.75, *p* = 0.03, *η*^*2*^ = 0.07, but not the method effect, *F*(1, 66) = 1.09, *p* = 0.30, *η*^*2*^ = 0.02. Simple effects tests indicated that the two groups neither differed on the balance operation, *F*(1, 66) = 3.71, *p* = 0.06, partial η^2^ = 0.05, nor on the inverse operation, *F*(1, 66) = 0.13, *p* = 0.72, partial η^2^ = 0.00.

The third question in the concept test required students to judge whether a pair of equations was equivalent with respect to the ‘ = ‘ sign concept of the operational line, followed by rating the mental effort invested in judging the pair of equations.

A 2 (method) × 2 (concept) ANOVA on mental effort yielded a significant concept effect, *F*(1, 66) = 7.56, *p* = 0.01, partial η^2^ = 0.10 indicating that both groups invested lower mental effort when processing the inverse operation (4.33 *vs*. 4.47) than the balance operation (4.41 *vs*. 4.70). The method × concept interaction was nonsignificant, *F*(1, 66) = 1.91, *p* = 0.17, partial η^2^ = 0.03, nor was the method effect significant, *F*(1, 66) = 0.22, *p* = 0.64, partial η^2^ = 0.00.

In sum, as hypothesized, similar to Experiment 1 and Experiment 2, higher scores on the practice equations and the post-test corresponded to fewer operational and relational lines (i.e., equations with a positive pronumeral). In support of hypothesis 1 (ii), the balance group was inferior to the inverse group not only for the practice questions but also for equations with a negative pronumeral in the post-test. As hypothesized, the two groups did not differ on the concept test with respect to the relational line. In line with hypothesis 2 (ii) and hypothesis 3, both groups performed better on the inverse operation than the balance operation and invested lower mental effort when judging the inverse operation than the balance operation. This evidence provides a degree of validation of the differential level of element interactivity in favour of the inverse operation.

## General discussion

The study of appropriate pedagogical methods for effective learning is an important topical theme in the field of educational psychology. Our conceptualization for investigation, reflected in three experimental undertakings, emphasized two contrasting pedagogical methods, which educators use in teaching and learning of linear equations: balance *vs*. inverse. We applied the balance method and the inverse method to acquire conceptual knowledge and procedural knowledge on learning to solve linear equations that varied in levels of element interactivity. Overall, from the three experiments, evidence ascertained makes empirical and theoretical contributions, which advance our understanding of the operational nature of different pedagogical methods, and the impact of the level of element interactivity upon learning.

### Empirical contributions

Results from the first two experiments, in general, indicate that the acquisition of procedural knowledge favoured one-step and two-step equations involving low rather than high level of element interactivity, irrespective of whether it was the balance group or the inverse group (i.e., hypothesis 1 (i)). Differential level of element interactivity within the one-step equations was reasonably substantial enough to cause the inverse group to gain better understanding of the procedural knowledge on practice equations than the balance group (i.e., hypothesis 1 (ii)). The inverse group, in contrast, had no advantage over the balance group in relation to the learning of two-step equations. It seems that a difference of one extra relational line between the two types of two-step equations was insufficient to result in differential acquisition of procedural knowledge.

Experiment 3, expanding on the first two experiments, focused on the learning of one-step and two-step equations with a positive pronumeral (low element interactivity) and a negative pronumeral (high element interactivity). Higher performance on equations with a positive pronumeral was observed for both the balance group and the inverse group (hypothesis 1 (i)). Because of the greater differential level of element interactivity within one-step and two-step equations, the inverse group demonstrated better understanding of procedural knowledge than the balance group, not only for the practice equations but also the equations with a negative pronumeral in the post-test (hypothesis 1 (ii)).

Concerning the acquisition of conceptual knowledge for linear equations, the results were consistent across the three experiments. The inverse group was as efficient as the balance group in interpreting the quantitative relation between the right side of the equation and the left side with respect to the relational line (hypothesis 2 (i)). Both groups performed better on the inverse operation, which in this case, imposed lower level of element interactivity than the balance operation (hypothesis 2 (ii)). We speculate that perhaps, the balance group realized, for example, the redundancy of the “+3–3” on the left side of the equation and, consequently, chose to ignore this. In other words, the balance group may interpret, for example, *y* + 3–3 = 10–3 as *y* = 10–3. More importantly, in Experiment 3, both the balance group and the inverse group invested lower mental effort when judging the inverse operation, which provided evidence of the hypothesized differential level element interactivity favouring the inverse operation (i.e., hypothesis 3).

### Theoretical considerations

Different level of element interactivity between the balance method and the inverse method depends not only on the type of operation (balance *vs*. inverse) on each operational line but also the number of operational and relational lines. The inverse operation imposes a lower level of element interactivity for each operational line, as compared with the balance operation. Nevertheless, the inverse method did not have any advantage over the balance method unless the differential level of element interactivity within the linear equations was substantially large enough. Overall then, the results from our investigation are consistent with previous research, suggesting that the superiority of one instruction over another instruction is manifested under the condition in which there is an adequate gap between low and high element interactivity material [12, 54]. Moreover, our results also pointed to the negative learning effect, consequently as a result of using an instruction (i.e., balance method) that increases the intrinsic cognitive load of the material.

The flexibility of applying two inverse operations concurrently for linear equations with a negative pronumeral reduces the number of operational and relational lines and therefore the level of element interactivity. Moreover, it also eliminates the need for students to operate with a negative pronumeral and a negative number. On this basis, we contend that the inverse method provides students with a means to counter the inherent difficulty of learning to solve linear equations that involve a negative pronumeral and negative numbers.

The classification of linear equations based on the number of operational and relational lines would enable mathematics educators to plan the teaching and learning of linear equations in a hierarchical order of complexity [28]. Consequently, the learning of a more complex equation (e.g., two-step equation) can be built on the prior knowledge of a simpler equation (one-step equation), thus reducing the burden on working memory load.

### Applied educational practices for consideration

A recent study by Ding [55] reveals that both U.S. and China primary mathematic textbooks highlight the opportunities to learn inverse operations. For example, primary school students learn that 4 + 3 = 7 is the same as 3 = 7–4 or 4 = 7–3. Here, the focus is the interplay between addition (e.g., + 4) as an inverse operation to subtraction (e.g.,– 4) in the context of an equation. To go a step further, we can ask students to compare 4 + 3 = 7 and *x* + 2 = 8. If they can understand 4 + 3 = 7 is the same as 4 = 7–3; then, they may understand *x* + 2 = 8 is the same as *x* = 8–2. As pointed out by Ding [55], without adequate foundation of the inverse operation, students may not be able to master the topic of differentiation and integration in calculus, functions and inverse functions in algebra in senior mathematics curriculum.

Our findings indicate that the inverse group was not disadvantaged regarding the acquisition of conceptual knowledge for linear equations. We noted that the mean proportion scores of the inverse group on the balance operation across the three experiments were above 50%. This implies then that more than half of the students in the inverse group could comprehend the balance operation, despite the fact that they did not have access to it. From this observed contention, the notion that the inverse method may fall short of addressing the conceptual knowledge of equation solving is not substantiated. Accordingly, capitalizing the inverse method for learning of linear equations could potentially help middle school students to make a smooth transition to senior mathematics in the future.

**Fig 6:**
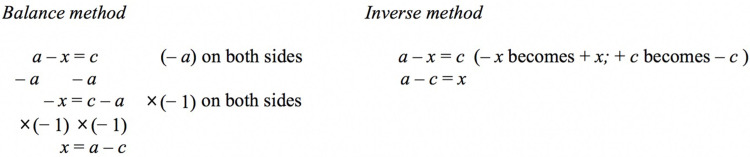
The balance method and inverse method to solve an algebra transformation equation.

The transformation of empirical results into educational practice is an important issue for consideration. For example, a comparison between the balance method and the inverse method reveals that the inverse method is particularly useful for algebra transformation problems with a negative variable such as *a*–*x* = *c*, solve for *x* (Fig 6). Using the inverse method, we can apply two inverse operations concurrently to solve for *x* (i.e., *a–c = x*), resulting in having one operational line and one relational line in the solution procedure. In contrast, using the balance method, the solution procedure will involve more operational lines (2 *vs*. 1) and relational lines (3 *vs*. 1) than the inverse method. Obviously, the solution procedure of the balance method looks cumbersome and error prone because it involves the manipulation of a negative variable.

On this basis, what can we conclude from our experimental undertakings? Our findings reinforce the potential of the inverse method to assist learners, in general. We contend that, in summary, incorporating the inverse method in mathematics textbooks *can benefit student learning of linear equations*, which may involve a negative pronumeral and a negative number.

### Caveats and future directions

Asquith, Stephens [50] coded “the same as” to indicate relational understanding of the ‘ = ‘ sign concept in an equation. However, we coded both “the same as” and “equal to” as correct answers. We reasoned that students’ understanding of the ‘ = ‘sign concept in terms of quantitative relationship between the right side and left side of the equation includes: (i) the right side is the same as the left side–“the same as”, and (ii) the right side is equal to the left side–“equal to”. Nonetheless, Alibali, Knuth [56] coded “it is equal to” as unspecified equal–that is, student could have interpreted “equal to” as to compute the answer. To ensure that we evaluate students’ relational understanding of the ‘ = ‘ sign concept accurately, additional research could ask students to give a reason after they have provided answers such as “the same as” or “equal to”.

We could include other types of two-step equations, which consist of more operational and relational lines to increase the gap between the low and high element interactivity knowledge for the two-step equations. For example, in regard to the two-step equation of 2 –*n*)/3 = 6, the balance method and the inverse method share the same three operational lines but differ in the number of relational line (5 *vs*. 4). Moreover, there is also room to further increase the gap between the one-step and two-step equations with a positive pronumeral and a negative pronumeral by including, for example, (2 –*n*)/3 = 6. Thus, if time permits, we encourage researchers and educators to include a wide range of linear equations with a positive pronumeral and a negative pronumeral.

In line with cognitive load research [12], the acquisition phase in which students studied an instruction sheet and completed a set of acquisition equations would expect to facilitate learning of linear equations. Nevertheless, we could have also invited a teacher to introduce linear equations using the materials in the instruction sheet, which may further enhance the learning outcomes across the balance method and the inverse method. For example, differential performance outcomes on one-step linear equations (Experiment 1) in favour of the inverse group may manifest not only for the practice problems but also the post-test. Therefore, it would be of interest to include a teaching phase in future enquiry.

The main purpose of Experiment 3 was to increase the gap between two types of one-step and two-step equations (a positive pronumeral *vs*. a negative pronumeral) in terms of the level of element interactivity. Differential level of element interactivity favours the inverse method because of two factors: (1) lower level of element interactivity associated with the inverse operation, and (2) perform two inverse operations concurrently reduces the number of relational and operational lines. Moreover, manipulating a positive pronumeral and positive numbers as a result of performing two concurrent inverse operations may also contribute to greater learning outcomes of the inverse method. Overall, we argue that an advantage of the inverse method over the balance method was mainly due to the use of two concurrent inverse operations. Nevertheless, future research could repeat Experiment 3 using the inverse operation sequentially in order to ascertain the benefit of using two concurrent inverse operations for linear equations with a negative pronumeral.

Given that the rationale of using the inverse operation sequentially or concurrently is similar, one may query whether knowing how to use the inverse operation sequentially in Experiments 1 and 2 could have also contributed to students’ understanding of using two inverse operations concurrently in Experiment 3. To address the issue, additional research could test the effect of learner levels of expertise in relation to sequential use of the inverse operation upon their understanding of using two inverse operations concurrently for linear equations.

Owing to the characteristics that underline human cognitive architecture, the complexity of materials makes little sense without considering learner levels of expertise. In fact, it has been suggested that the *expertise reversal effect* [19] is a variant of the element interactivity effect [18]. The expertise reversal effect occurs when an instruction that is effective for novices becomes less effective and eventually ineffective when they gain expertise in the domain. This is because the beneficial information for novices becomes redundant for experts.

Differential level of element interactivity not only arises from varying degrees of complexity of materials, but also from the changes in learner levels of expertise. For example, experienced learners who have acquired a lower-level schema for one-step equations (e.g., 2*x* = 6), the learning of a higher-level schema of two-step equations (e.g., 5*x* + 2 = 12) would require the learning of the first two lines only (i.e., 5*x* + 2 = 12, 5*x* = 12–2). This is because the rest of the solution procedure is similar to the solution procedure of one-step equation, 2*x* = 6. Therefore, differential level of element interactivity in terms of the solution procedure of linear equations exists between novice learners and experienced learners. Research has documented the positive effect of *prior knowledge of Algebra* on learning to solve linear equations [57]. Students who had prior knowledge of Algebra tended to outperform those who did not. Hence, we could explore the relationships between pedagogical methods (e.g., balance *vs*. inverse), learner levels of expertise in linear equations, and the complexity of linear equations in future research.

Overall, it should be noted that the strength of element interactivity depends not only on the complexity of materials, but also learner levels of expertise. Currently, we cannot accurately measure levels of element interactivity. As this stage, we can only estimate levels of element interactivity given that we can only estimate learner levels of expertise.

## Conclusion

In conclusion, the results of the three experiments are promising, suggesting that the inverse method may serve as an alternative for the solving difficult linear equations that have a negative pronumeral and negative numbers. Moreover, aside from this testament, the three experimental studies also verify the importance of the concept of element interactivity, which could operate to discern the complexity of a particular instructional method.

## Supporting information

S1 Appendix (DOCX)
