# Supplementary material for: Advancing the study of solving linear equations with negative pronumerals: A smarter way from a cognitive load perspective
Source: PLoS One. 2022 Mar 18;17(3):e0265547. doi: 10.1371/journal.pone.0265547 (PMC8932579; doi:10.1371/journal.pone.0265547)
Supplement: S1 Appendix — (DOCX) [file pone.0265547.s001.docx]

### Appendix A

*Solution Procedure of Linear Equations with a Negative Pronumeral*

Balance method Inverse method

Experiment 1: One-step equations

| 8 – *a* = 13 (– 8) on both sides  – 8 + 8 – *a* = 13 – 8  – *a* = 5 [note: – *a* = – 1) *a*]  $\frac{-a}{-1}=\frac{5}{-1}$ ÷ (– 1) on both sides  *a* = – 5 | 8 – *a* = 13 (+ 8 becomes – 8)  – *a* = 13 – 8  – *a* = 5 [note: – *a* = (– 1) *a*] $a=\frac{5}{-1}$ (– 1) becomes ÷ (– 1)  *a* = – 5  *Note: apply inverse operation sequentially* |
| --- | --- |

Experiment 2: Two-step equations

| 22 – 5*p* = 2 (– 22) on both sides  – 22 + 22 – 5*p* = 2 – 22  – 5*p* = –20 ÷ (– 5) on both sides  $\frac{-5p}{-5}=\frac{-20}{-5}$  *p* = 4 | 22 – 5*p* = 2 (+ 22 becomes – 22)  – 5*p* = 2 – 22  – 5*p* = – 20 ((– 5) becomes ÷ (– 5)  $p=\frac{-20}{-5}$  *p* = 4  *Note: apply inverse operation sequentially* |
| --- | --- |

Experiment 3: One-step and two-step equations

| *One-step equation*  11 – *x* = 7 (– 11 on both sides)  –11 + 11 – *x* = 7 –11  – *x* = – 4 [note: – *x* = (– 1)*x*]  $\frac{-x}{-1}=\frac{-4}{-1}$ (÷ (– 1) on both sides)  *x* = 4 | 11 – *x* = 7 (– *x* becomes + *x*)  (+ 7 becomes – 7)  – 7 + 11 = *x*  4 = *x*  *Note: apply two inverse operations* concurrently |
| --- | --- |

| *Two-step equation*  5 – 3*t* = – 19 (– 5 on both sides)  – 5 + 5 – 3*t* = – 19 – 5  – 3*t* = – 24 [note: – 3*t* = (– 3)*t*]  $\frac{-3t}{-3}=\frac{-24}{-3}$ ÷ (– 3) on both sides  *t* = 8 | 5 – 3*t* = –19 (– 3*t* becomes + 3*t*)  (–19 becomes + 19)  +19 + 5 = 3*t*  24 = 3*t* (3 becomes ÷ 3)  $\frac{24}{3}=t$  8 = *t*  *Note: apply two inverse operations concurrently* |
| --- | --- |

### Appendix B

*Sample of worked examples*

*Balance method*

**Exercise (If you need help, you may ask a question)**

Study each worked example carefully and ensure that you understand it because the following equation that you are going to solve will be similar. You must show your working in the space provided.

***Section 1***

| Worked example 1  *p* + 9 = 11 (- 9 on both sides)  *p* + 9 – 9 = 11– 9  *p* = 2  Equation 1  *y* + 7 = 21 | Worked example 2  *m* – 5 = – 7 (+ 5 on both sides)  *m* – 5 + 5 = – 7 + 5  *m* = – 2  Equation 2  *x*– 3 = 9 |
| --- | --- |

*Inverse method*

**Exercise (If you need help, you may ask a question)**

Study each worked example carefully and ensure that you understand it because the following equation that you are going to solve will be similar. You must show your working in the space provided.

***Section 1***

| Worked example 1  *p* + 9 = 11 (+ 9 becomes -9)  *p* = 11– 9  *p* = 2  Equation 1  *y* + 7 = 21 | Worked example 1  *m* – 5 = – 7 (- 5 becomes + 5)  *m* = – 7 + 5  *m* = – 2  Equation 2  *x*– 3 = 9 |
| --- | --- |

### Appendix C

*Samples of Post-test*

|  | One operational + two relational lines | Two operational + three relational lines |
| --- | --- | --- |
| One-step equations | 1.  – 9 = 4 2. = 7 | 1. – 4 –*y* = 5 2.  |
|  | Two operational + three relational lines | Two operational + four relational lines |
| Two-step equations | 1. 3*m* – 1 = 5  2. ****= 6 | 1. = 4 |
|  | Equation with a positive pronumeral | Equation with a negative pronumeral |
| One-step and two-step equations | 1. *a* + 8 = 17 2. 5*n* – 2 = 38 | 1. 10 – *t* = 0  2. 4 – 5*t* = –1 |

Note: There is only one type of two-step equation that has two operational and four relational line.

### Appendix D

Samples of Concept Test

Question 1

*x* + 6 = 11

The arrow points to a symbol. What does the symbol mean?

Question 2

Are the equation pairs below equivalent? (circle the answer)

| Experiment 1: One-step equations | | |
| --- | --- | --- |
| 1. *x* + 3 = 5 2. *x* + 3 = 5 | *x* + 3 – 3 = 5 – 3  *x* = 5 – 3 | Yes No  Yes No |
| Experiment 2: Two-step equations | | |
| 1. = 6 2. = 6 |  2= 6 2  = 6 2 | Yes No  Yes No |
| Experiment 3: One-step and two-step equations  Equation with a positive pronumeral | | |
| (a) *p* – 7 = 11  (b) 2*x* + 6 = 5 | *p* = 11+ 7  2*x* + 6 – 6 = 5 – 6 | Yes No  Yes No |

Equation with a negative pronumeral

| (a) 8 – *y =* 0  (b) 5 – 4*n* = –1 | 8 – 0 = *y*  –5 + 5 – 4*n* = – 1 – 5 | Yes No  Yes No |
| --- | --- | --- |

*Note:* Question 1 was common across Experiments 1, 2 and 3. For Question 2, samples of equation pairs were listed here.
